# Wind alters plant-pollinator community structure, bee foraging rate & movements between plants

**DOI:** 10.1093/beheco/araf067

**Published:** 2025-06-15

**Authors:** Nicholas J Balfour, Francis L W Ratnieks

**Affiliations:** Laboratory of Apiculture & Social Insects, Department of Evolution and Ecology, School of Life Sciences, Biology Road, University of Sussex, Brighton, BN1 9QG, United Kingdom; Laboratory of Apiculture & Social Insects, Department of Evolution and Ecology, School of Life Sciences, Biology Road, University of Sussex, Brighton, BN1 9QG, United Kingdom

**Keywords:** abiotic factors, foraging ecology, pollinators, wind

## Abstract

Wind is an important abiotic factor that influences an array of biological processes including animal behavior, but it is rarely considered in plant–pollinator interactions. Here, we investigate the impact of wind speed on plant-pollinator community structure under natural conditions. In our field experiment we observed threefold greater abundance of pollinators and double the species richness under low (< 0.5 m/s) versus moderate (> 2.5 m/s) wind speeds. Significant differences in plant-pollinator community structure were also observed, with Diptera most abundant under low wind conditions, and Hymenoptera predominant and Lepidoptera absent in windier conditions. Across three plant species, the foraging rates of both honey and bumble bees were 15% greater at low versus moderate wind speeds. Lastly, at greater wind speeds bees made more movements between plants, suggesting that wind may improve yield for crops that require cross-pollination. Overall, our findings show that wind is an important factor in determining which pollinator groups are active and that eusocial bees, which are often the most important pollinators, were little affected by moderate wind speeds. Our results raise several important issues for further study and suggest that the use of linear features such as trees and hedges that act as windbreaks may be beneficial to crop pollination and conservation efforts.

## Teaser

Wind influences an array of biological processes including animal behavior, but it is rarely considered in pollinator ecology. Our field study shows that even moderate wind speeds alter plant-pollinator communities. We observed a threefold greater abundance of pollinators and double the number of species under low versus moderate wind speeds. Significant changes in community structure were also seen across wind speeds. Thus, these findings have important implications for both crop pollination and pollinator conservation efforts.

## Introduction

Wind can affect flying animals via both airflow and turbulence (Crall et al. 2017;  Matthews and Sponberg 2018), potentially increasing the energetic cost of an already expensive mode of locomotion and introducing further problems such as loss of control. From purely physical considerations these challenges should be greater for insects than birds or bats, due to insects’ smaller body sizes resulting in greater surface to volume ratios (Hunter 2007). Wind can affect insect behavior and ecology in multiple contexts including migration (Drake and Farrow 1988), mating and courtship (Marchand and McNeil 2000), as well as interactions with plants including herbivory (eg Paré and Tumlinson 1999; Yamazaki 2011) and flower visitation (eg Hennessy et al. 2020, 2021).

Both pollinator behavior and ecology are known to be affected by wind. For example, butterflies have been observed to vacate more exposed habitats as wind speed increases (Dover et al. 1997) and flower visitation by flies is severely hampered at greater wind speed (Inouye et al. 2015). Honey bee, *Apis mellifera*, and solitary bee (*Osmia cornuta*) foraging activity decreases at greater wind speeds in commercial apple orchards (eg Vicens and Bosch 2000). Euglossine bee energy expenditure has been shown to increase by 30% under windy conditions (Combes and Dudley 2009). Flower visitation is also indirectly affected, as flowers sway in windy conditions making landing and take-off potentially more difficult (Hennessy et al. 2021).

Although insects are relatively small animals, they vary greatly in body size. Flower-visiting insects range from large moths such as *Manduca sexta* (2.6 g; Wasserthal 2001 ) and bumble bees queens (1 g; Owen 1988), to small solitary bees such as *Perdita minima*; (0.333 mg; Capinera 2008). Parasitoid wasps can be even smaller, for example *Encarsia formosa* (0.015mg; Sane 2016). Thus, bees range over more than three orders of magnitude in mass and flower-visiting insects over five or more. Moreover, some insects such as butterflies have relatively large wings for their mass, and so may be more affected by wind.

Insects also vary greatly in their need to visit flowers. The majority of insects are either predatory or herbivorous during their larval stage and often carry reserves over to their adult phase, so only visit flowers as adults to meet their own energetic requirements. Worker bees and most female solitary bees, however, mainly forage to provision their brood, which are non-mobile and fed by adults (Balfour et al. 2018). These physical, morphological and life-history factors lead to the general hypothesis that wind should differentially affect the various groups of flower-visitors, with large bees predicted to be least affected due to their greater body mass, relatively smaller wings (ie in comparison to Lepidoptera) and need to feed brood.

Here we investigate the impact of wind speed on British plant-pollinator community structure, bee foraging rate and movements between plants under natural conditions. In doing so we address, in part, a significant knowledge gap in the community ecology of this economically and biologically important mutualism (Kearns and Inouye 1993;  Primack and Inouye 1993; Crall et al. 2020).

## Methods

### Pollinator censuses

To determine the abundance and species richness of flower-visiting insects across a range of wind speeds we studied five common and widely distributed native plant species that attract a range of pollinator species: (i) *Achillea millefolium* (common yarrow), (ii) *Centaurea nigra* (black knapweed), (iii) *Chamaenerion angustifolium* (rosebay willowherb), (iv) *Cirsium vulgare* (spear thistle), and (v) *Senecio jacobaea* (common ragwort). We observed patches of flowers during ~30 five-minute censuses, per species. Data were collected between 1000 to 1600 h on 15 study days, from 13 July to 16 August 2023 and 9 July to 23 August 2024 at five sites in Edinburgh, UK: (i) Arthur’s Seat (55.94581, -3.16407); (ii) The Hermitage of Braid and Blackford Hill Local Nature Reserve (55.92028, -3.19671); (iii) Craigmillar Castle Park (55.92718, -3.14720); (iv) Bruntsfield Links (55.93960, -3.19705); and (v) Easter Craiglockhart Hill Local Nature Reserve (55.91993, -3.22975).

Flower patches were located by walking from the approximate center of each study site at a randomly generated compass angle. Patches ≤ 5m to either side of the observer, in full-flower, and with ≥ 25% cover of the target species within a 1m^2^ quadrat were studied. First, a 1m^2^ quadrat was placed over the part of the patch with the greatest number of flowers. We then estimated: (*i*) the percentage ground cover of the study plant species within the quadrat (cover), using 10% intervals; and (*ii*) the area (m^2^) of the study species within a 3m radius (patch area), using 1m^2^ intervals. All flower-visitors observed actively foraging on the patch were then recorded in situ for 5 min. The observer was positioned downwind from the patch when possible. We also recorded both (i) wind speed (using the anemometer “average” function, which gives the mean wind speed over the previous 10 s) and (ii) temperature at 1.5m at the beginning and end of each census using an anemometer and thermometer (BTMETER BT-100, sensitivity: 0.1m/s and 0.1°C). The mean was calculated from the two wind speeds (mean wind speed) and two temperatures (mean temperature) recorded per census. Insects were identified to species or assigned a morphospecies name in situ and from a distance that did not alter their behavior. Care was taken not to record the same individual more than once per sampling occasion. The minimum distance between two consecutively studied patches was 10m to reduce the likelihood of individual pollinators being recorded across multiple patches.

### Foraging observations

Data were collected between 1000 to 1600 h during five days, from 08 August 2023 to 21 September 2023 and 04 June 2024 to 03 September 2024 at three sites in Edinburgh, UK: (i) Arthur’s Seat; (ii) The Hermitage of Braid and Blackford Hill Local Nature Reserve; and (iii) Craigmillar Castle Park.

We quantified the number of *C. angustifolium*, *Echium vulgare* (viper’s bugloss) and *Pentaglottis sempervirens* (green alkanet) flowers visited (foraging rate) during 1 min for two or three of the following bee species: (i) *Apis mellifera*, (ii) *Bombus pascuorum*, and (iii) *B. terrestris*. We recorded both the (*i*) wind speed and (*ii*) temperature at the beginning and end of each census (see section above for further details). Lastly, we quantified the abundance and diversity of pollinators foraging on our study patches at the beginning and end of each data collection period for *C. angustifolium* (*n* = 24), *Echium vulgare* (n = 20) and *P. sempervirens* (*n* = 22).

We simultaneously collected data on inflorescence switch rate (ie number of flowers visited/number of inflorescences visited) for the two or three bee species across the three plant species.

### Statistical analysis

Statistical analyses were conducted in “R” software (version 4.3.2; R-Project 2024). To analyze data we used Generalised Linear Mixed Models (GLMMs). Explanatory variables were selected using a forward stepwise selection process. The model with the lowest Akaike Information Criterion score was selected using the R package bbmle (Brooks et al. 2017). Likelihood ratio tests provided p values for explanatory variables. Model assumptions were checked visually using the R package DHARMa (Hartig and Lohse 2022).

The response variables (species richness and abundance) from our pollinator censuses were analyzed separately. The global models for these two analyses contained five explanatory variables: time of day, proportion cover (logit transformed), patch area, mean wind speed, flower species, and mean temperature, and two random variables: study location and date. Both models used a Poisson error distribution. The final species richness model included one explanatory variable (mean wind speed) and both random variables. The final abundance model included four explanatory variables (mean wind speed, proportion cover, patch area, and flower species) and both random variables.

The response variables (foraging rate and inflorescence switch rate) from our foraging observation data were analyzed separately. The global models for these two analyses contained two explanatory variables: mean wind speed and bee species, and four random variables: flower species, mean temperature, study location and date. The interaction between mean wind speed and flower species was also included in the foraging rate model as a random variable. The response variable for the inflorescence switch rate model was logit transformed prior to analysis, and the model used a Gaussian distribution. The foraging speed model used a negative binomial distribution. Both final models included all explanatory and random variables.

**Fig. 1.**
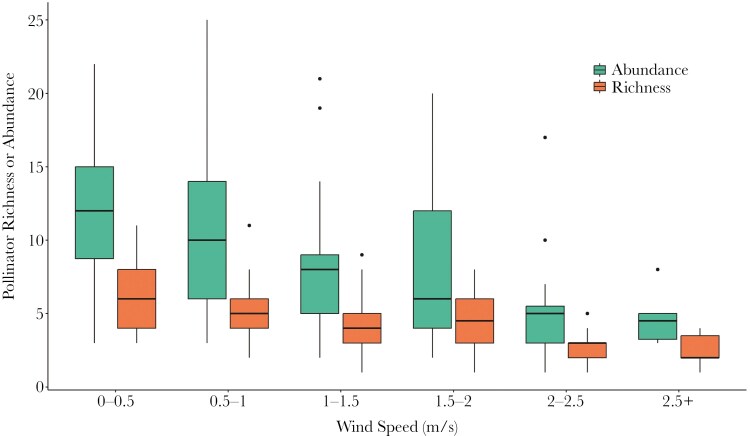
Foraging pollinator abundance (number of individuals, green) and species richness (number of species, orange) from 148 five-minute censuses on five plant species combined across a range of wind speeds (recorded at a height of 1.5m). Box plots show median and interquartile range, whiskers standard error and circles outliers.

**Fig. 2.**
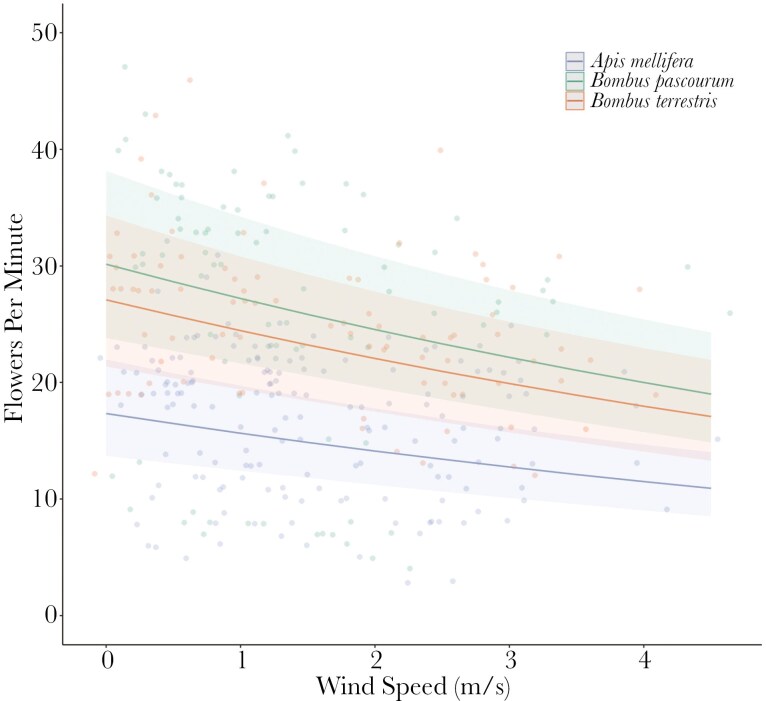
Relationship between foraging rate (flowers visited per minute) and wind speed for three bee species (green: Apis mellifera, red: Bombus pascourum, blue: B. terrestris) foraging on three plant species: rosebay willowherb (*Chamaeleon angustifolium*), green alkanet (*Pentaglottissempervirens*), and viper’s bugloss (*Echium vulgare*). Shown are predicted regression lines, 95% Confidence Intervals (shaded areas) and raw data points (circles, n = 330).

The ggpredict function from the R package ggeffects (Lüdecke 2018).to generate model predictions and confidence intervals for the foraging rate and proportion of movements between inflorescences models (Figs 1 and 2).

## Results

### Pollinator censuses

We recorded 1278 pollinators across 148 censuses. Hymenoptera (56.0%) were the most recorded order, followed by Diptera (34.0%), Coleoptera (9.7%), and Lepidoptera (0.3%). The five most commonly recorded, and identified, species were *Apis mellifera* (honey bees; 22.9%), followed by *Bombus terrestris* (buff-tailed bumble bee; 11.7%), *Bombus pascuorum* (common carder bee 9.8%), *Rhagonycha fulva* (common red soldier beetle 9.5%), and *Episyrphus balteatus* (marmalade hoverfly; 6.1%).

Pollinator abundance (2.94-fold greater) and species richness (2.5-fold greater) were several fold greater at low (0 to 0.5 m/s) than moderate (> 2.5 m/s) wind speeds. Pollinator species richness per census was significantly negatively related to wind speed (*χ*^2^ = 28.24, *P* < 0.001; Fig. 1a; Table 1). Pollinator abundance per census and was also significantly negatively associated with wind speed (*χ*^2^ = 38.44, *P* < 0.001; Fig. 1b; Table 1), positively associated with proportion cover (*χ*^2^ = 9.72, *P* = 0.002) and significantly affected by flower species (*χ*^2^ = 41.46, *P* < 0.001).

**Table 1.** Output of generalized linear mixed models. As the data were overdispersed, had a quasi-Poisson error distribution. Likelihood ratio tests provided p values for fixed factors.

	Predictor variable	Coefficient	Standard error	z value	P
**Insect**	Wind	-0.316	0.122	-6.376	<0.001
**abundance**	Area	0.031	0.027	1.136	0.256
	Cover	0.146	0.046	3.158	0.002
	*Centaurea nigra*	0.233	0.096	2.420	0.016
	*Chamaenerion angustifolium* *Cirsium vulgare* *Senecio jacobaea*	-0.453 -0.067 -0.022	0.108 0.108 0.103	-4.211 -0.620 -0.218	<0.001 0.536 0.827
**Insect richness**	Wind	-0.338	0.064	-5.257	<0.001

### Foraging observations

We collected 330 foraging rate observations across three bee species foraging on three plant species. The number of flowers visited per minute was found to be significantly negatively related to average wind speed (*χ*^2^ = 35.55, P < 0.001; Fig. 2). This trend was consistent across all species and the variable ‘bee species’ was found to be significant in this model (*χ*^2^ = 211.2, *P* < 0.001). The interaction between wind speed and flower species was found to not significantly impact the number of flowers visited per minute (*χ*^2^ < 0.001, *P* = 0.98). Across the three plant and bee species the number of flowers visited per minute was 15% greater at the lowest (0 to 0.5 m/s) than the highest (> 2.5 m/s) wind speed category.

Honey bees (53%) were the most common species observed foraging on *C. angustifolium*, followed by *B. pascuorum* (24%), *B. terrestris* (20%) and the occasional lepidopteran (3%). Honey bees (65%) were the most common species observed visiting *P. sempervirens*, followed by *B. pratorum* (18%), *B. pascuorum* (10%), *B. terrestris* (6%), and *B. lapidarius* (1%). Honey bees (58%) were also the most common species observed visiting *E. vulgare*, followed by *B. terrestris* (27%), *B. hypnorum* (12%), *B. pascuorum* (11%), and *B. soroeensis* (2%).

**Fig. 3.**
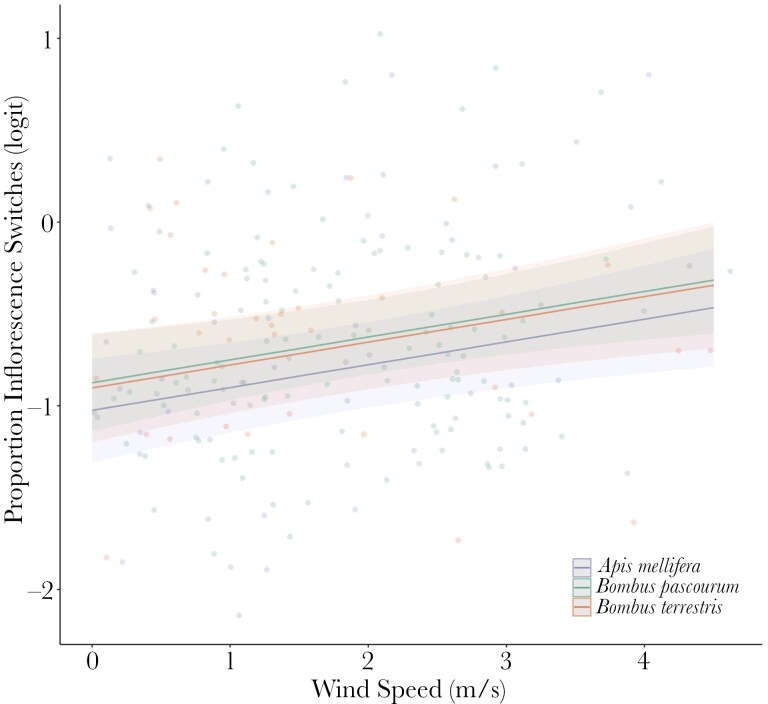
Relationship between the proportion of inflorescence switches (number of movements between inflorescences/ number of flowers visited per minute) and wind speed for three bee species (green: Apis mellifera, red: Bombus pascourum, blue: B. terrestris) foraging on three plant species: rosebay willowherb (Chamaeleon angustifolium), green alkanet (Pentaglottis sempervirens), and viper bugloss (Echium vulgare). Shown are predicted regression lines, 95% Confidence Intervals (shaded areas) and raw data points (circles, n = 211).

We collected 211 observations of inflorescence switches across three bee species and three plant species. All observed inflorescence switches were to another inflorescence of the same plant species. The proportion of switches between inflorescence per minute was significantly positively related to average wind speed (*χ*^2^ = 8.01, *P* = 0.005; Fig. 3). This trend was consistent across bee species studied. The variable ‘bee species’ was not significant (*χ*^2^ = 2.30, *P* = 0.317).

## Discussion

Our results clearly show that a moderate increase in wind speed plays an important role in determining plant-pollinator community structure. Insect pollinator abundance, species richness and the foraging rate of bees were all greater at low than at moderate wind speeds.

**Fig. 4.**
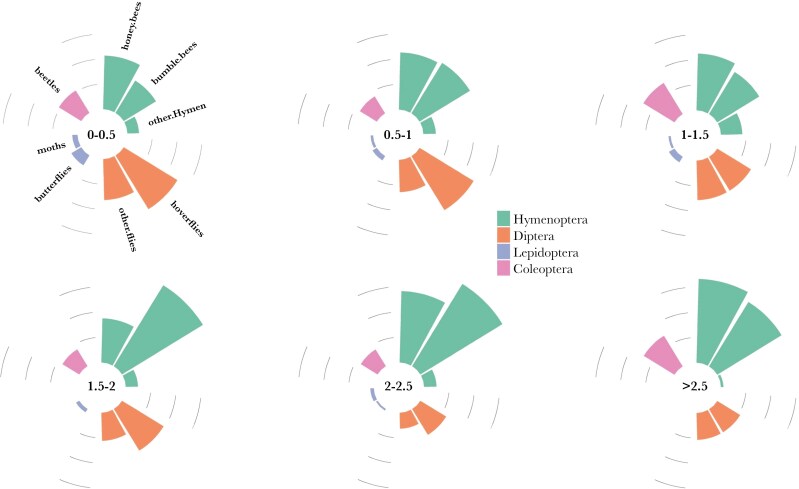
Proportion of foraging insect groups per order (green: Hymenoptera, orange: Diptera, blue: Lepidoptera, purple: Coleoptera) foraging on five plant species across six wind speeds intervals (given in the centre of each circle in meters per second), recorded at a height of 1.5m. Concentric circles represent 10%, 20%, and 30%.

We recorded three times more (194%) pollinators and twice the species richness (125%) under low (< 0.5 m/s) versus moderate (> 2.5 m/s) wind speeds. At low wind speeds Diptera were the most species-rich group constituting nearly half of all species recorded (49%). However, at the higher, but still moderate wind speeds (> 2.5 m/s), Hymenoptera dominated (73% of all species) with Diptera richness being reduced (22%). Lepidoptera were rarely recorded at low wind speeds (0.5% of species) and unrecorded under moderate conditions (Fig. 4). This may be due to the wing-mass ratio of butterflies, which exceeds that of other insects (Suzuki et al. 2019). Conversely, Coleoptera appeared unaffected by wind speed. This order is known to be the slowest foraging group of flower visitors (Couvillon et al 2015). In our study Coleoptera were dominated by one species (common red soldier beetle, *R. fulva*), which is often observed in large mating aggregations on flowers, where adults use nectar and pollen resources and move little.

The foraging rate of honey bees and bumble bees significantly decreased with higher wind speeds. This result was consistent across all study plant species, with bees visiting on average 15% more flowers in still and low wind conditions (< 0.5 m/s) than at moderate wind speeds (> 2.5 m/s).

As predicted, larger eusocial bees, honey bees and bumble bees, were the pollinator group least affected by increasing wind speeds. We hypothesize that this is due to their greater body mass and need to forage for their colony (Balfour et al. 2018;  Balfour et al. 2021). How does wind impact the energetic returns for these bees? Our data show that bees visit 15% fewer flowers at moderate wind speeds. However, our results also show that greater wind speeds lead to fewer competitors and presumably, therefore, greater intervals between flower visits by all insects. Thus, assuming wind does not impact nectar secretion rates (eg Silva et al. 2004), the standing crop and energetic reward per flower may actually be greater under windy conditions. As such, winds of 2.5 m/s may have little or no effect on energetic returns for pollinators and may even increase returns for those that are active.

To put the wind speeds recorded in this study into context, the terrestrial global average is approximately 3.0 to 3.5 m/s (Zeng et al. 2019). However, these measurements are collected at a standard height of 10m, as specified by the World Meteorological Organisation. Wind speed at this height is approximately 2.5 times greater than at the height (1.5 m) we gathered our data (Balfour et al., in prep.), which is ecologically relevant to pollinators visiting flowers on herbaceous plants. Our lowest wind speed category is equivalent to Beaufort Scale 0 to 1 (calm) and our highest category (2.5 to 4 m/s) is equivalent to Beaufort Scale 4 to 5 (moderate to fresh breeze). After several decades of global “stilling,” terrestrial wind speeds have been increasing since 2010 (Zeng et al. 2019). Future climate predictions for wind speeds are mixed, but they are projected to increase during the second half of this century (Lowe et al. 2018). Hence, our data indicate that future climate change may pose an additional and hitherto underappreciated challenge for pollinating insects.

We recorded three times more pollinators (2.94) and bees visited 15% more flowers (1.15) when winds were low (Table 2). Therefore, our results imply that more than three times (3.38) as many flowers are visited per unit time under still than windy conditions. These findings strongly suggest that the use of linear features such as trees and hedges that act as windbreaks in horticultural cropland would benefit crop pollination. As Diptera foraging activity was acutely curtailed by higher wind speeds, the use of windbreaks may be particularly beneficial for crops for which flies play an important role as pollinators.

**Table 2.** Summary of pollinator census data per wind speed category: mean ± SE wind speed, mean ± SE pollinator abundance, and mean ± SE pollinator species richness recorded.

Wind Speed Category (m/s)	Wind Speed (m/s)	Abundance	Species Richness
0 to 0.5	0.36 ± 0.12	11.96 ± 0.86	6.25 ± 0.47
0.5 to 1	0.72 ± 0.11	10.48 ± 0.74	5.30 ± 0.40
1 to 1.5	1.31 ± 0.15	7.97 ± 0.69	4.41 ± 0.38
1.5 to 2	1.79 ± 0.16	8.00 ± 0.75	4.28 ± 0.41
2 to2.5	2.18 ± 0.11	5.63 ± 1.06	2.81 ± 0.58
>2.5	3.07 ± 0.14	4.06 ± 0.94	2.50 ± 0.44

However, for crops that require cross-pollination (eg apples, *Malus domestica* and almonds, *Prunus dulcis*), our data indicate that greater wind speeds may increase the number of movements between plants or trees and thus improve fruit set. Interspecific interactions between bees are also known to increase movement between plants (Brittian et al. 2013). Our results indicate that the frequency of these interactions will be reduced at greater wind speeds.

Our study shows the important effects of wind on plant-pollinator community structure and the foraging rate of honey bees and bumble bees. But, it also raises several important questions that are central to the ecology of plant-pollinator mutualism and which merit future research. One important question is the effect of increasing wind speed on pollinator foraging energetics. Is exploitative competition for nectar reduced (eg Balfour et al. 2015), possibly enhancing energy gain for those insects that remain active? Within this same area of bee and pollinator foraging energetics, how does wind speed affect nectar secretion and concentration, if evaporation is facilitated by wind? The second major question is pollination efficiency. By reducing the foraging rate, will higher wind speeds reduce pollination efficiency? Or will it increase pollination efficiency by causing bees to move more often between plants? Most plants require cross-pollination, and a little buffeting by wind may actually enhance insect pollination.

## Data Availability

Analyses reported in this article can be reproduced using the data provided by Balfour and Ratnieks (2025).
